# Optimization and test of pneumatic lifting seed distributor for maize plot seeder

**DOI:** 10.3389/fpls.2025.1469963

**Published:** 2025-03-17

**Authors:** Qingbin Song, Pengfei Zhao, Yubin Bi, Zuoli Fu, Yuxiang Huang, Yi Zheng

**Affiliations:** ^1^ College of Mechanical and Electronic Engineering, Northwest A&F University, Yangling, China; ^2^ Shaanxi Engineering Research Center for Agricultural Equipment, Northwest A&F University, Yangling, China; ^3^ Qingdao Eplot Machinery Co., Ltd., Qingdao, China

**Keywords:** agricultural machinery, plot seeding, pneumatic lifting, seed distributing uniformity, structural parameter

## Abstract

**Introduction:**

The seed distributors are the core components of the plot seeder. Their functions are to distribute the seeds evenly to the seed metering device corresponding to each sowing row. In order to solve the problem of uneven seed distributing of the seed distributor of maize plot seeder under tilted state, this study designed a pneumatic lifting seed distributor and optimized its structure and working parameters.

**Methods:**

A theoretical analysis of the seed distributing process was carried out. The sliding, rolling and bouncing states of maize seeds on the cone were analyzed. The parameters’ range that affected the seed distributing uniformity of the seed distributor were determined through single-factor tests. A three-factor three-level Box-Behnken design (BBD) test was carried out with cone height, seed storage tube diameter and seed storage tube lifting height as the test factors.

**Results:**

It was concluded that sliding down along the cone surface was the best movement way of seed distributing. The BBD test results showed that cone height, seed storage tube diameter and seed storage tube lifting height had highly significant effects on the variation coefficients of seed distributing uniformity in the three situations that the seed distributor were horizontally tilted at 0° vertically tilted at 0°, horizontally tilted at 5° vertically tilted at 0°, and horizontally tilted at 5° vertically tilted at 5°. The optimal structural and operational parameter combination of the seed distributor were as follows: cone height of 77 mm, seed storage tube diameter of 70 mm, and seed storage tube lifting height of 42 mm. Under this parameter combination, the bench test was carried out to verify that the variation coefficient of seed distributing uniformity were 5.13%, 7.07%, and 6.54% when the seed distributor were in three tilted states. The test results were basically consistent with the predicted values. The variation coefficient of seed distributing uniformity in field test was 1.23 percentage points lower than that of the traditional seed distributor.

**Discussion:**

The pneumatic lifting seed distributor has better seed distributing effect than traditional seed distributor.The research provided a technical reference for the optimization and improvement of the seed distributor of the plot seeder.

## 1 Introduction

The overall level of mechanization in China’s seed industry is much lower than that of field production, and the development of mechanization in the seed industry faces many problems and challenges (Zhu et al., 2015). The seed distributors are the key component of the plot seeder for uniform portion seeding, which can realize one time seed supply and uniform seed distributing for multiple rows. The Oyjord seed distributor was the first to realize portion sowing in the drill, which was recognized by relevant researchers, and has been used to until now (Shang et al., 2021). The plot seeders produced by companies such as Wintersteiger, Haldrup, Almaco and others all apply the principle of the Oyjord seed distributor.

The seed distributors used in plot drills differ significantly from those used in field drills. Pneumatic centralized seed distributors are commonly used in field drills (Lei et al., 2018; Mudarisov et al., 2020; Hu et al., 2021). While traditional plot seed distributors include cone centrifugal type (Yang et al., 2015; Cheng et al., 2019), cone belt type (Yang et al., 2016), and cone grid disc type (Yu et al., 2023). The seed distributor of the plot drill can realize the two functions of seed distributing and seed metering at the same time. Researchers have conducted many studies on the seed distributors of the plot drill. Zhang et al. (2017) combined the traditional cone-belt seeding device with an electronically controlled automation system to achieve a periodic self-cleaning time-series conversion seed distributing operation. Liu et al. (2010, 2011a, 2011b) studied the effects of the forward speed of the plot drill, the rotation speed of the seed filling device, the cone generatrix length of the seed filling device and the cone base angle of the seed filling device on the variation coefficient of seed distributing uniformity and the variation coefficient of seed filling uniformity of five varieties including rape and wheat. Yang et al. (2013) investigated the sliding movement of forage seeds along the cone generatrix on the seeding cone and the rotational movement around the axis of the seeding cone. For precision seeders, there is no need to distribute seeds when sowing in the field. The seeds can be sent to the seed metering device through air flow or mechanical devices (Gao et al., 2022, 2023; Zhao et al., 2024). Therefore, it is not suitable for plot precision seeders. Researchers have conducted some research on plot precision seeders (Yang et al., 2019a; Dun et al., 2022). However, the design of the seed distributors were mainly based on the production practice experience of the plot drill, and lacks design bases (Shang et al., 2021). The existing plot precision seeder seed distributors are mainly cone uniform grid. Yang (2019b) optimized the seed distributing performance of the cone uniform grid seed distributor in a horizontal state through multi-factor tests.

The above studies were all conducted when the seed distributor was in a horizontal position. However, when the ground is uneven during actual operation, the seed distributor will tilt, which affects the seed distributing uniformity (Shang et al., 2021). The existing solution is to level the seed distributor through a leveling mechanism (Yang, 2019b). Nevertheless, the seeding process is fast and the leveling response made by manual or system has a certain lag, which cannot ensure that the seed distributor can return to a horizontal state in time (Song, 2019). Therefore, in actual operation, more seeds than the set sowing amount are often prepared. The reason is to ensure that there are enough seed populations in the seed filling area of the seed metering device to prevent large-scale missed seeding. If the seed distribution of the seed distributor is not even, it will also cause seriously missed seeding in a certain row and insufficient seeding amount. After sowing, the remaining seeds in the seed metering device will be discarded. The problem of uneven seed distribution results in the waste of seeds (Shang et al, 2010). In summary, existing researches on the seed distributors of plot precision seeders are insufficient. The design and optimization of the seed distributor were only carried out under the horizontal condition of the seed distributor, and no solution was proposed to ensure good seed distributing uniformity when the seed distributors was tilted. In order to solve the above problems, this study optimized the structural and operational parameters of the seed distributor through simulation and bench tests based on the traditional cone uniform grid seed distributor. The optimized seed distributor had good seed distributing uniformity within the tilt angle range of 0~5°, which improved the seed distributing uniformity of the seed distributor of the maize plot precision seeder.

## 2 Materials and methods

### 2.1 The structure of the pneumatic lifting seed distributor

#### 2.1.1 Overall structure

**Figure 1:**
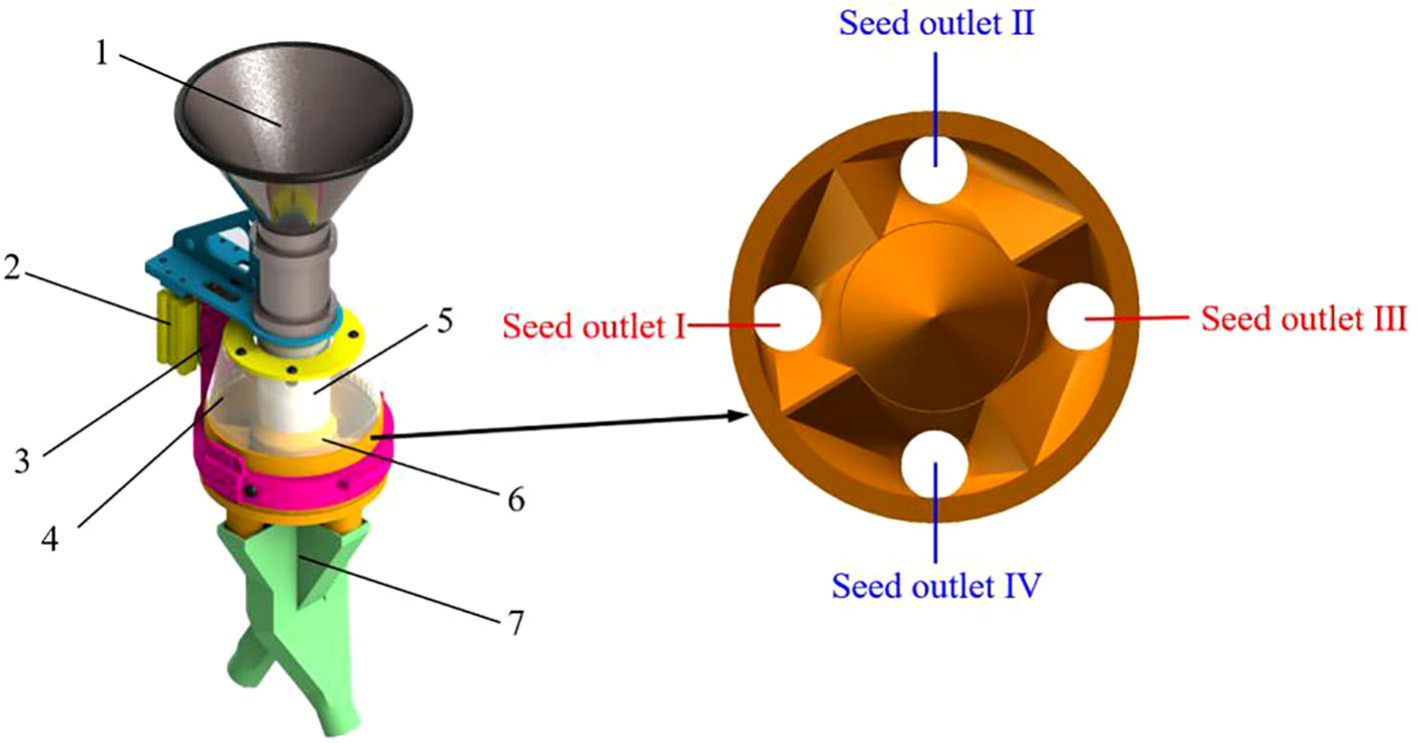
Schematic diagram of the structure of the pneumatic lifting seed distributor. 1. Seed throwing funnel; 2. Pneumatic lifting mechanism; 3. Air cylinder fixing seat; 4. Transparent shield; 5. Seed storage tube; 6. Cone uniform grid mechanism; 7. Y-shaped seed guiding tube.

The pneumatic lifting seed distributor is an important component of the maize plot precision seeder. Its structure is mainly composed of a seed throwing funnel, a pneumatic lifting mechanism, a air cylinder fixing seat, a transparent shield, a seed storage tube, a cone uniform grid mechanism and a Y-shaped seed guiding tube, as shown in **Figure 1**. The seed throwing funnel is fixedly connected to the seed storage tube, which can transport maize seeds to the seed storage tube. The seed storage tube and the conical surface of the cone uniform grid mechanism fit tightly to form a seed storage space. The cone uniform grid mechanism is mainly composed of a cone and four grids. The four grids are symmetrically distributed along the center, dividing the bottom area of the cone into four parts evenly. The diagonal grids are connected to the same Y-shaped seed guiding tube to receive seeds falling from the uniform grid seed outlet. The transparent shield is installed above the cone uniform grid to prevent the seeds from being ejected out of the seed distributor after colliding with the cone. The pneumatic lifting mechanism provides power for lifting and resetting the seed storage tube. The air cylinder of the pneumatic lifting mechanism is installed on the air cylinder fixing seat.

#### 2.1.2 Design of pneumatic lifting mechanism

**Figure 2:**
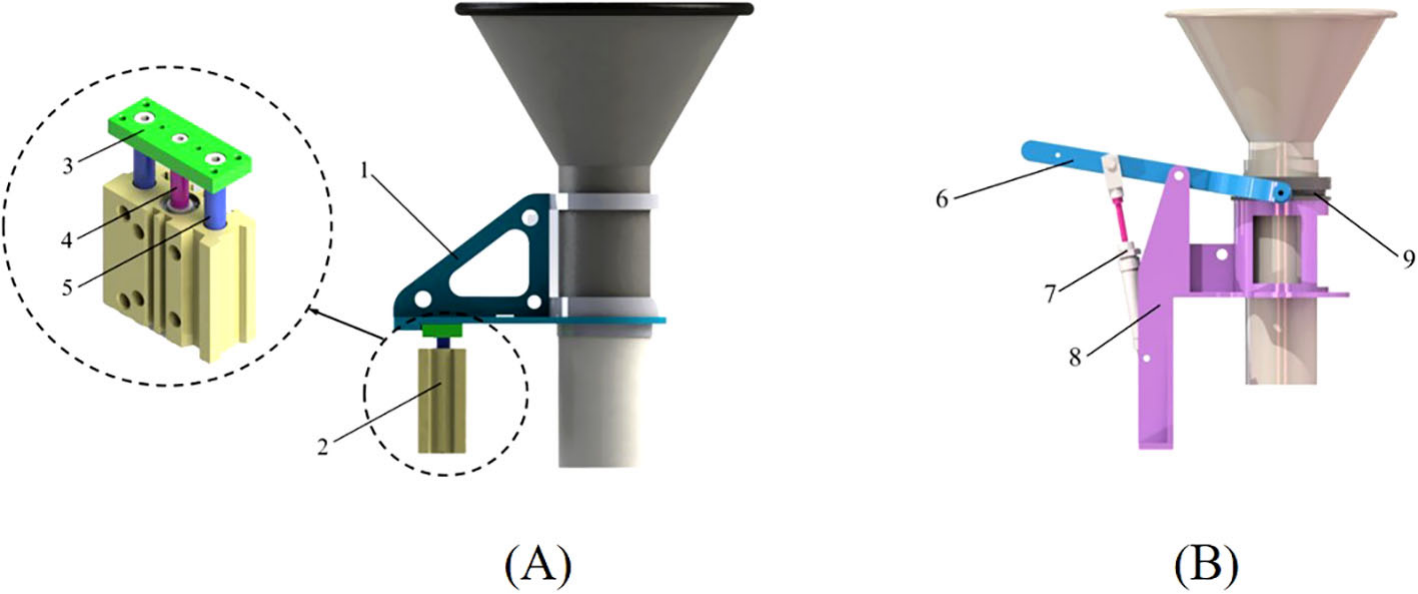
The structure of lifting mechanism. **(A)** Pneumatic lifting type; **(B)** Traditional lever type. 1. Connector between seed storage tube and air cylinder; 2. Three-axis cylinder; 3. Baffle; 4. Piston rod; 5. Guide rod; 6. Articulated lever 7. Single-axis cylinder 8. Fixed bracket 9. Limiting groove.

The traditional seed storage tube lifting mechanism usually drove the seed storage tube to complete the lifting and resetting actions through an articulated lever. One end of the lever was hinged to the pneumatic device, the other end was hinged to the seed storage tube, and the middle was hinged to the fixed bracket. The articulated lever structure had an angle change during the rotation process. Therefore, a limiting groove was provided on the traditional seed storage tube to ensure that the seed storage tube moved in the vertical direction. However, the changes in the limiting groove and the inclination angle caused the traditional seed storage tube lifting device to bear greater friction. In actual production, there was a jamming problem with the lifting device. This affected the lifting accuracy of the seed storage tube, resulting in poor seed distribution effect. In view of the above problems, this study proposed a method in which a air cylinder directly drove the movement of the seed storage tube and designed a new pneumatic lifting mechanism, the structure of which is shown in **Figure 2**. The designed pneumatic lifting mechanism was based on the traditional pneumatic lifting mechanism, but removed the middle lever and replaced it with a connecting piece that moved with a single degree of freedom. By reducing the transmission process between components, the pneumatic lifting mechanism directly drove the connecting part to move in the vertical direction through the piston rod and the guide rod. The connecting piece was fixedly connected to the seed storage tube, thereby realizing the lifting and resetting of the seed storage tube. The pneumatic lifting seed distributor designed in this paper can ensure that the seeder had good seed distributing uniformity in a tilted state without the need to add an additional leveling mechanism. The improved lifting device avoided the problem of the seed storage tube being stuck when being lifted. A comparison of these two devices is shown in **Table 1**.

**Table 1:** Comparison of seed distributors.

Comparison items	Type of seed distributor	
	Pneumatic lifting	Traditional type
Structure	Simple structure	Additional leveling mechanism required
Reliability	Smooth operation of the mechanism	Mechanism is prone to jamming
Leveling method	No leveling required	Manual leveling
Work efficiency	Higher	Lower

The seed storage tube was connected to the seed throwing funnel. During the seed distributing operation, the seed throwing funnel will move along the vertical direction with the seed storage tube. The total weight of the two is about 1 kg, that is, the axial load force on the air cylinder should be the gravity *G_Z_* of the seed throwing funnel and the seed storage tube, which is about 9.8 N. After comprehensively considering the installation size, a thin three-axis adjustable air cylinder with model MGPMJ20×50-30 was selected. The main parameters of the air cylinder are: air cylinder diameter 20 mm, piston rod diameter 10 mm, air cylinder stroke 30~50 mm adjustable, and working pressure 0.15~1.0 MPa. The air cylinder can provide 98 N of thrust *F_T_*. It fulfilled the requirements for seed storage tube lifting and resetting.

### 2.2 Operating principle

**Figure 3:**
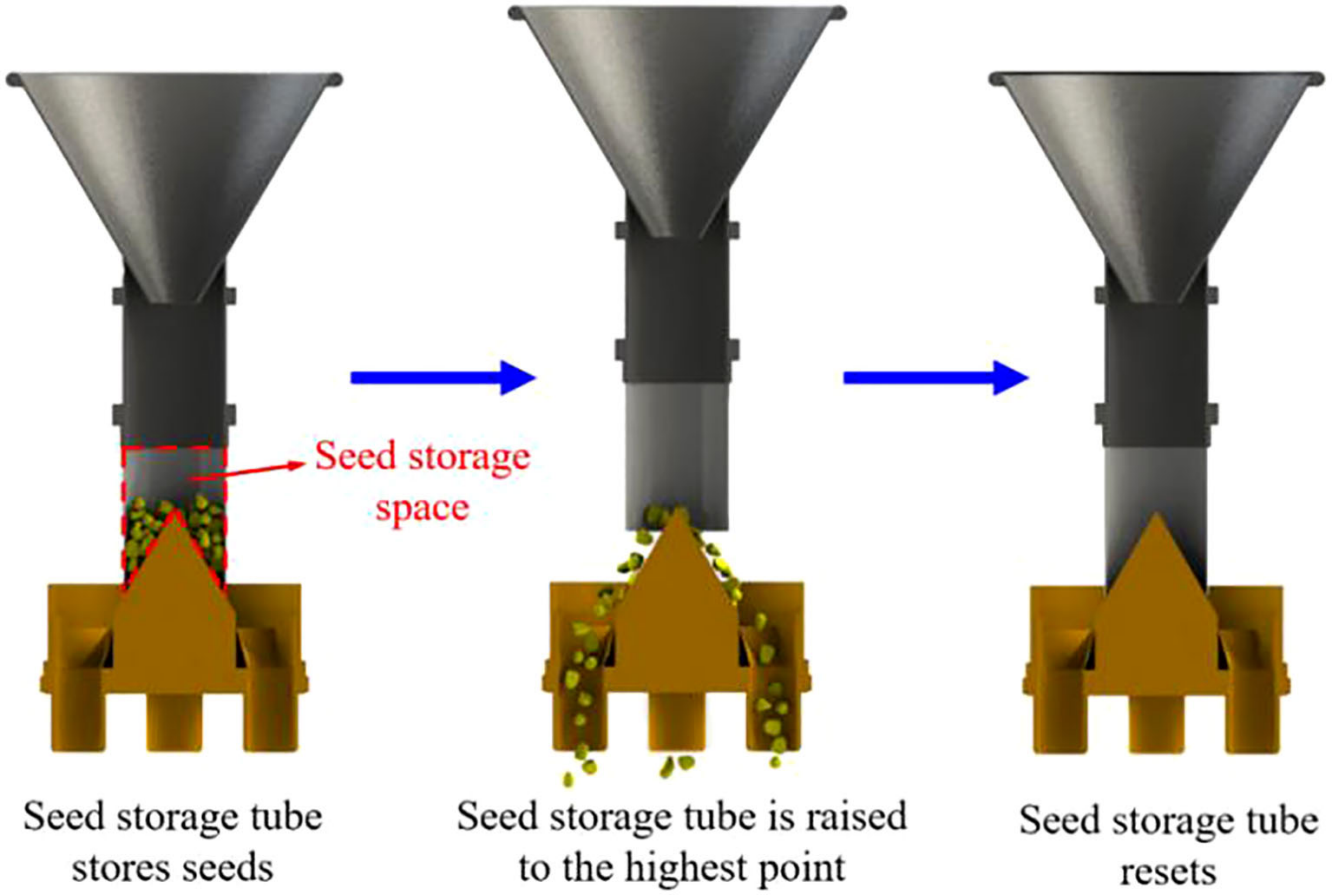
Schematic diagram of the movement process of the seed storage tube.

The maize seeds entered the seed storage tube through the seed throwing funnel, and the seeds fell under the action of their own gravity. They were pre-distributed through the cone and evenly distributed in the seed storage space formed by the close fit between the seed storage tube and the cone surface. The seed storage tube was lifted instantaneously under the drive of the pneumatic lifting mechanism. The movement process of the seed storage tube is shown in **Figure 3**.

Under the action of their own gravity, collision between seeds, friction between the cone surface and seeds, the seeds were distributed along the cone surface to the uniform grid at the bottom of the cone. The seeds evenly distributed by the uniform grid fell from their respective seed outlets and enter the Y-shaped seed guide tube. The seeds falling from outlets I and III entered the same Y-shaped seed guide tube. The seeds falling from outlets II and IV entered another Y-shaped seed guide tube. Finally, the seeds flow out from the outlet of the Y-shaped seed guide tube. Then the pneumatic lifting mechanism drove the seed storage tube back to the initial position to complete a single seed distributing operation.

### 2.3 Analysis of movement characteristics of maize seeds

The maize seeds can be divided into horse-tooth shape, spherical-cone shape and near-spherical shape according to their shapes. Among which horse-tooth shape accounts for the largest proportion (Dong et al., 2024; Markauskas et al., 2015; Zhou, 2021). In this study, the horse-tooth shape maize seed was taken as an example to analyze the forces of the seed on the cone, and establish the kinematic analysis model of the seed. The *xOy* coordinate system was established with the seed center of gravity as the origin *O* of the coordinate axis. The positive direction of the *x*-axis was downward along the cone surface, and the positive direction of the *y*-axis was rotated 90°counterclockwise from the *x*-axis. The maize seed existed on the cone in the state of sliding, rolling and bouncing. The maize seed on the cone surface were affected by the combined force of their own gravity *G*, the friction force *F_f_* of the cone surface and other forces.

**Figure 4:**
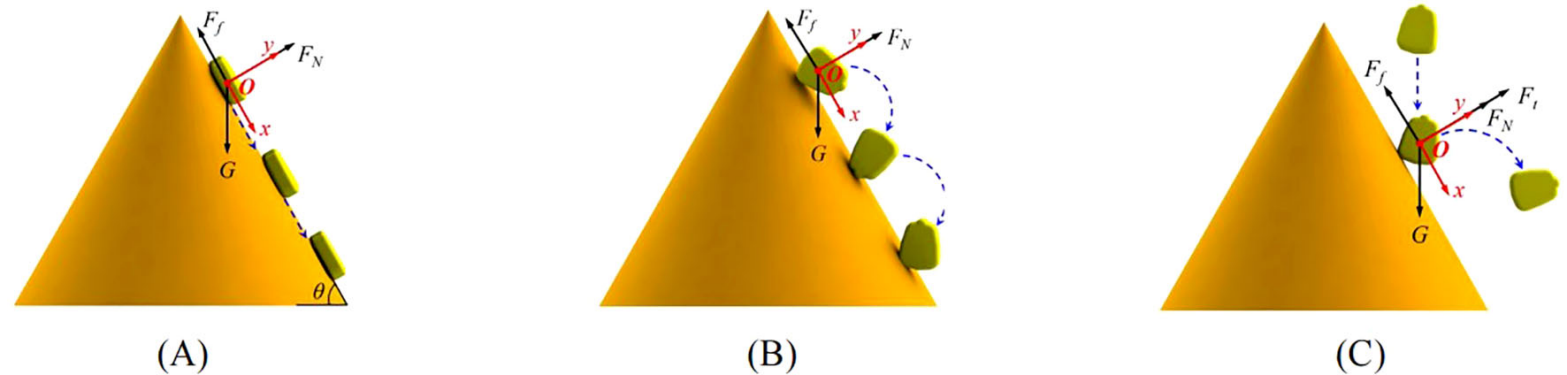
Force analysis of the maize seed. **(A)** Sliding; **(B)** Rolling; **(C)** Bouncing.


As shown in **Figures 4A, B**, the forces acting on the seed were basically the same when the seed slid and rolled on the cone surface. The force equations of the seed in the *x*-axis and *y*-axis directions when sliding and rolling are shown in Equation 1.

(1) $$\left\{ \begin{array}{l} \sum F_{x}=G\sin\theta -F_{f}=mg\sin\theta -F_{f}\\ \sum F_{y}=F_{N}-G\cos\theta =F_{N}-mg\cos\theta \end{array}\right.$$

Where *G* is the gravity force on the seed, (N); *F_N_* is the supporting force between the seed and the cone surface, (N); *F_f_* is the friction force between the seed and the cone surface, (N); m is themass of the seed, (kg); *g* is the acceleration of gravity, (m/s^2^); *θ* is the angle between the cone generatrix and the horizontal plane, (°).

Because the initial state of the seed and the contact point between the seed and the cone were different, two motion states of the seed occurred. When the initial state of the seed tended to be stationary, the seed would slide down under the action of *G*. When the initial state of the seed tended to rotate, *G* would generate a gravitational torque at this time. So that the seed would rotate close to the cone surface under the action of *G*. In this process, *F_N_* was always equal to *G*cos*θ*. If the seed leaf the cone surface, *F_N_* instantly became zero. Meanwhile the seed occurred to be out of the cone surface of the rotation.

The force analysis of the maize seed bouncing after colliding with the cone surface is shown in **Figure 4C**. Due to the limitation of the diameter of the seed storage tube, the seeds cannot completely contact the cone surface when they were in the seed storage space. When the seed storage tube was lifted, these seeds were only affected by *G* and falls freely. When the seeds collide with the cone surface, elastic force was generated at the moment of collision. At this time, the force model of the seeds is shown in Equation 2.


(2) $$\left\{ \begin{array}{l} \sum F_{x}=G\sin\theta -F_{f}=mg\sin\theta -F_{f}\\ \sum F_{y}=F_{t}+F_{N}-G\cos\theta =F_{t}+F_{N}-mg\cos\theta \end{array}\right.$$

Where *F_N_* is the elastic force on the seed, (N).

When the elastic force *F_t_* satisfied Equation 3, the maize seed bounced off the cone surface. The seed rotated as they detached from the cone surface, while continuing to move in a parabolic motion by *G* until it fell into the uniform grid.


(3) $$F_{t}>mg\cos\theta$$

The best movement state for the seed to be evenly distributed is when the seed slide down the cone surface. While the seed rolling and bouncing will change the best motion state of the seed, so that the seed falling into the uniform grid has greater uncertainty, resulting in poor seed distribution effect.

### 2.4 Establishment of simulation model

#### 2.4.1 Establishment of discrete element model for maize seeds

It is a common research method to study the influence laws between maize seeds and devices by building discrete element models (Gao et al., 2021). The test used maize seeds bred by Syngenta Group Seed Company, and the seeds were divided into three shapes: horse-tooth shape, spherical-cone shape and near-spherical shape, and the statistical proportion of the three shapes of seeds were 64%, 19%, and 17%, respectively. The three axial dimensions of the seeds (length, width, and thickness) were measured. The results showed that the length, width, and thickness of the maize seeds conformed to a normal distribution.

**Figure 5:**
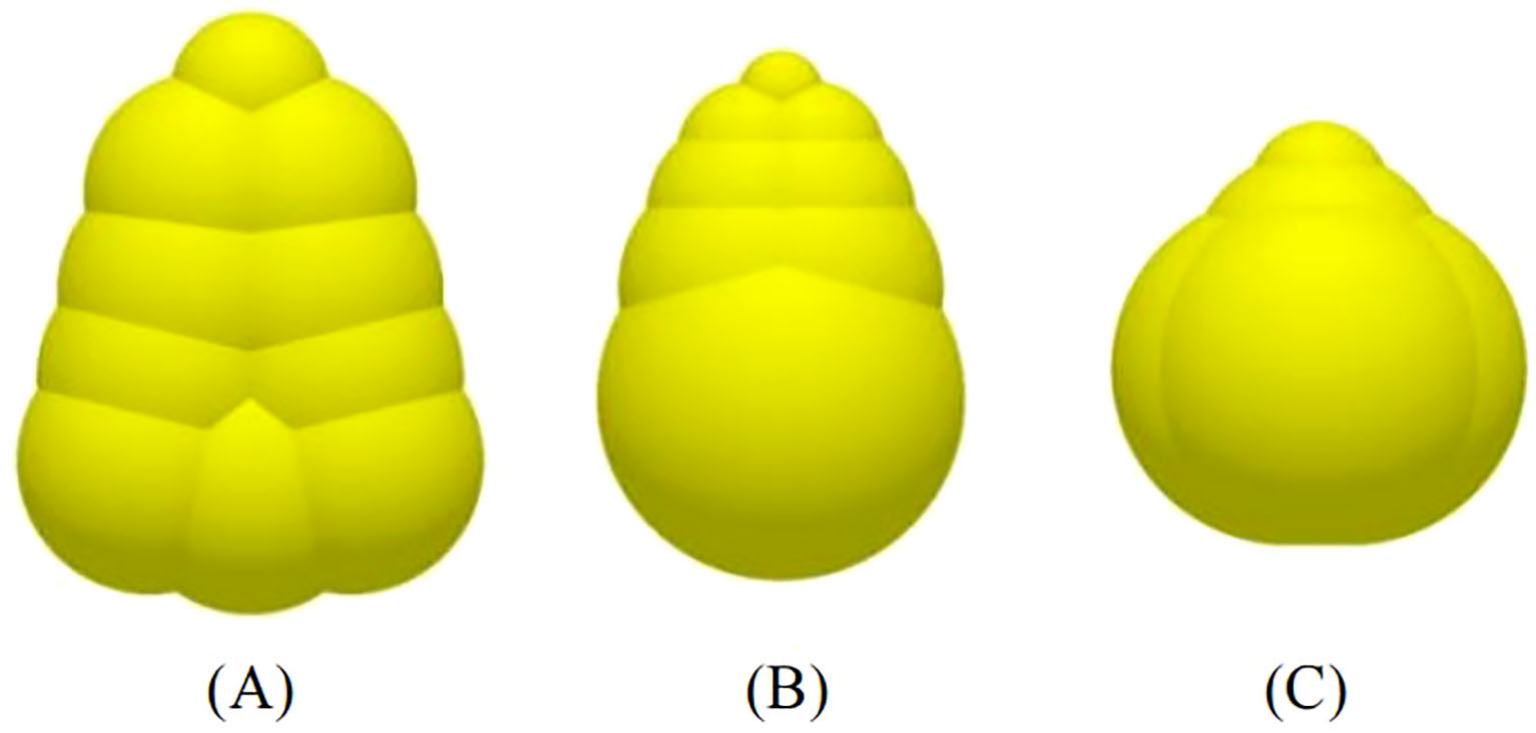
Discrete element model of maize seeds. **(A)** Horse-tooth shape; **(B)** Spherical-cone shape; **(C)** Near-spherical shape.

As shown in **Figure 5**, discrete elemental models of maize seeds in three shapes were built according to the three-axis dimension of the maize seeds (Markauska et al., 2015). In practical breeding tests, maize seeds have to be graded before sowing, and generally only seeds with widths between 6 mm and 9 mm in size are retained for sowing (Song, 2019). In actual production, usually 80 seeds are put into the seed distributor for each plot. To be more realistic, the particle factory was set up above the seed feeding funnel to generate 80 maize seed particles according to the actual proportion of three shapes of maize seeds. To ensure the accuracy of the simulation test, maize seed particles with widths ranging from 6 to 9 mm were generated by normal distribution.

#### 2.4.2 Setting simulation parameters

The seed throwing funnel, seed storage tube and cone uniform grid were all made of aluminum alloy, and the transparent cover was made of plexiglass. The simulation parameters were set according to the reference (Yang, 2019b), as shown in **Table 2**.

**Table 2:** Simulation parameters.

Parameter	Value
Poisson’s ratio of maize seed	0.4
Shear modulus of maize seed (Pa)	1.37×10^8^
Density of maize seed (kg/m^3^)	1197
Poisson’s ratio of aluminum alloy	0.25
Shear modulus of aluminum alloy (Pa)	2.70×10^10^
Density of aluminum alloy (kg/m^3^)	2700
Poisson’s ratio of plexiglass	0.5
Shear modulus of plexiglass (Pa)	1.77×10^8^
Density of plexiglass (kg/m^3^)	1180
Coefficient of restitution (seed to seed)	0.284
Coefficient of static friction (seed to seed)	0.342
Coefficient of rolling friction (seed to seed)	0.0545
Coefficient of restitution (seed to aluminum alloy)	0.709
Coefficient of static friction (seed to aluminum alloy)	0.342
Coefficient of rolling friction (seed to aluminum alloy)	0.0515
Coefficient of restitution (seed to plexiglass)	0.621
Coefficient of static friction (seed to plexiglass)	0.459
Coefficient of rolling friction (seed to plexiglass)	0.0931

#### 2.4.3 Establishment of discrete element model of seed distributor

**Figure 6:**
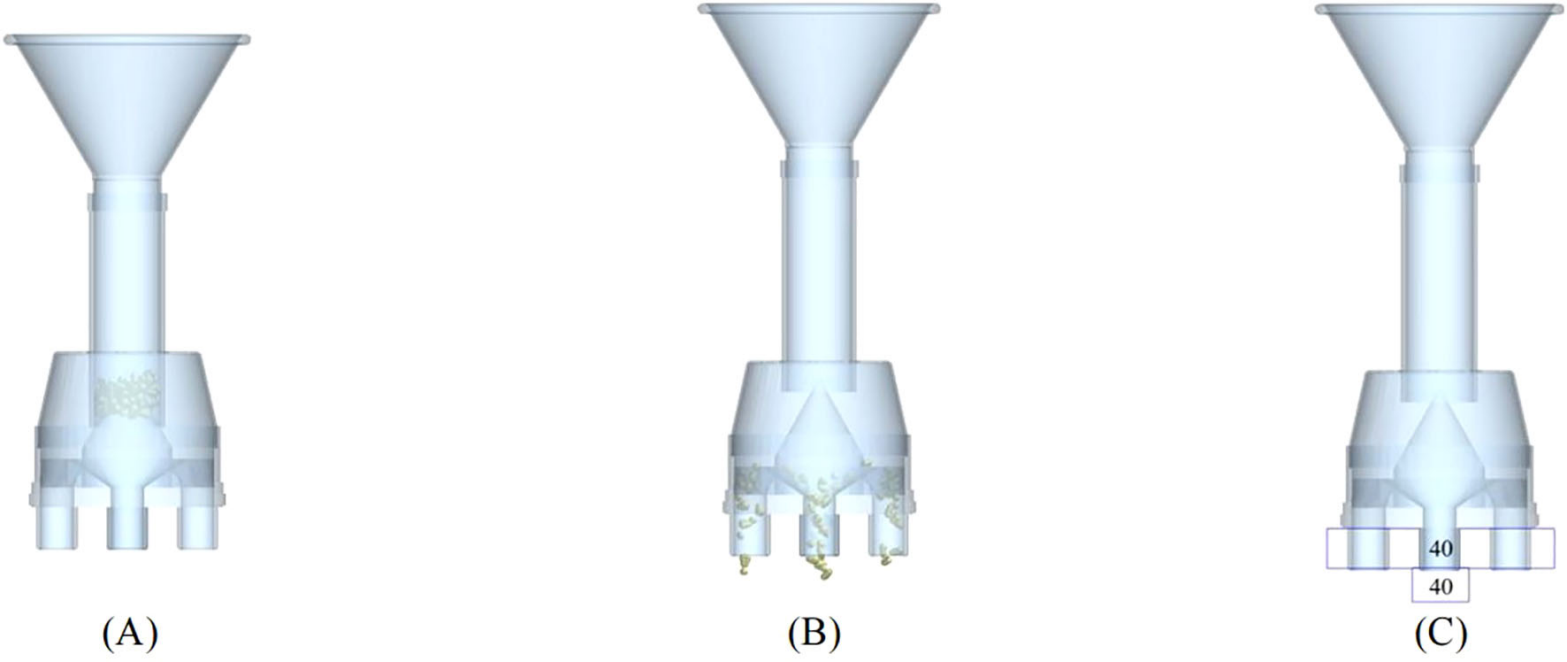
Simulation model of the seed distributor. **(A)** Before lifting the seed storage tube; **(B)** After lifting the seed storage tube; **(C)** Post-processing module.

The simplified three-dimensional model of pneumatic lifting seed distributor was established using three-dimensional modeling software. In order to save calculation time, only the key components of the seed distributor, such as seed throwing funnel, seed storage tube, cone uniform grid and transparent shield, were retained when it was imported into EDEM. The discrete element model of the seed distributor was shown in **Figure 6**. The post-processing module of EDEM was used to count the number of seeds falling in the diagonal grid.

### 2.5 Single factor simulation test

Under the condition of horizontal seed distributor, a single factor test was carried out with cone height *h*_1_, seed storage tube diameter *d* and seed storage tube lifting height *h*_2_ as the test factors. Through the single factor test, the influence law of each factor on the seed distributing performance of the seed distributor was explored. Each group of tests was repeated 20 times and the mean value was taken as the test result.

Since there was no relevant standard to formulate the test method and evaluation indicator for the seed distributor of maize plot seeders, this test selected the variation coefficient of seed distributing uniformity as the evaluation indicator based on the actual production requirements of maize plot seeders (Gong et al., 2011). The smaller the variation coefficient of seed distributing uniformity, the better the seed distributing effect. The variation coefficient of seed distributing uniformity is calculated by Equations 4 and 5.


(4) $$C=\frac{s}{\bar{x}}\times 100\%=\frac{\sqrt{\frac{\sum_{i=1}^{n}{\left(x_{i}-\bar{x}\right)}^{2}}{n-1}}}{\bar{x}}\times 100\%$$

(5) $$\bar{x}=\frac{\sum_{i=1}^{n}x_{i}}{n}$$

Where *C* is the variation coefficient of seed distributing uniformity between the Y-shaped seed guiding tubes, %; *s* is the standard deviation of the number of seeds; `*x* is the average number of seeds between each Y-shaped seed guiding tube; *x_i_* is the number of seeds in the *i*-th Y-shaped seed guiding tube; *n* is the number of Y-shaped seed guiding tubes.

### 2.6 Box-Behnken design test

**Figure 7:**
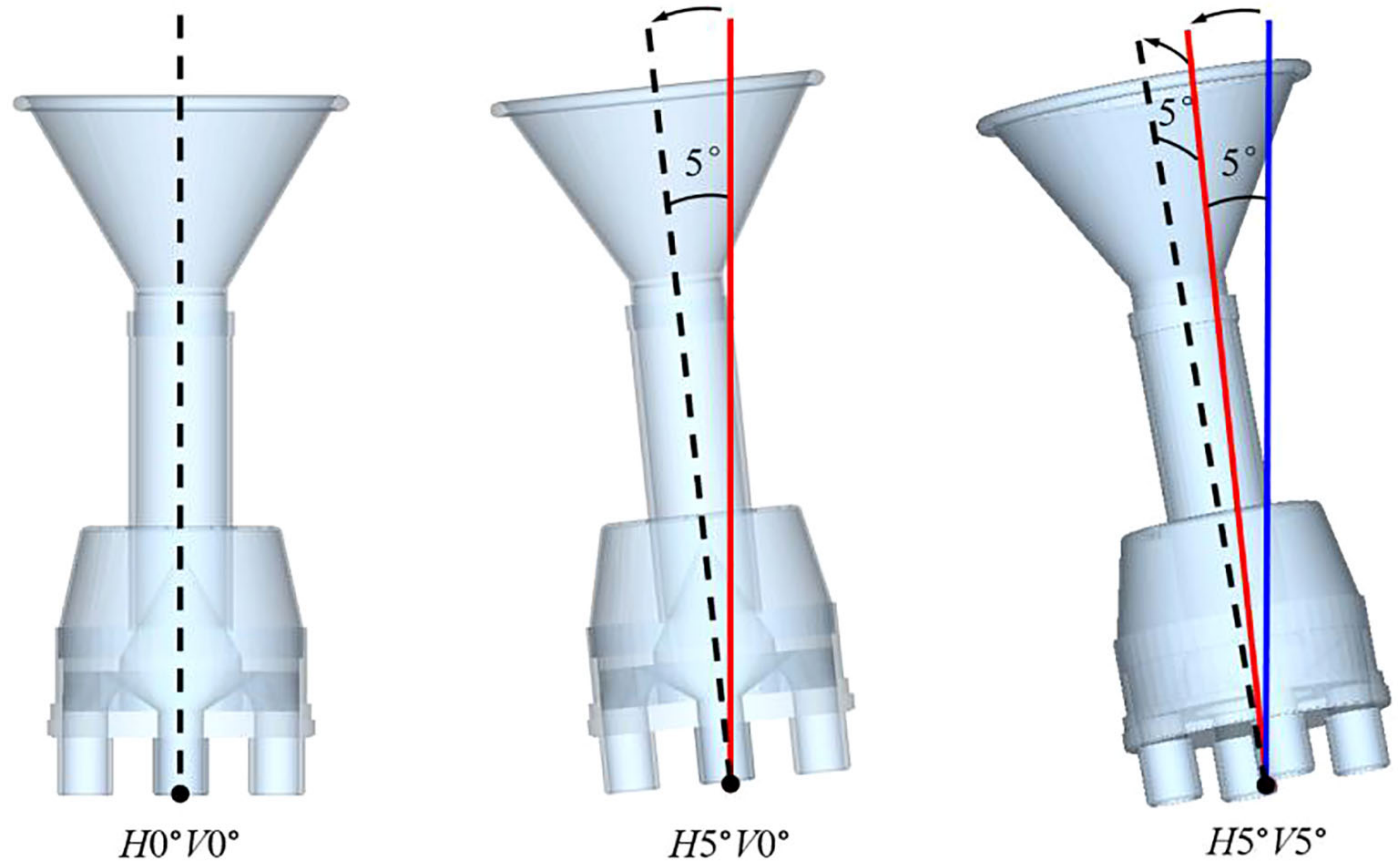
Schematic diagram of the tilting situation of seed distributor.

In actual production, the maize test plot is small and relatively flat, and the tilt angle of the plot seeder during operation generally does not exceed 5°. The results of the preliminary tests showed that the greater the inclination of the seed distributor, the worse the seed distributing uniformity. Therefore, as long as the seed distributor can ensure the seed distributing effect under extreme tilting conditions, the seed sorting effect can also be guaranteed under non-extreme conditions. Therefore, the selection of a tilt of 5° as the test factor is more representative. Since the cone uniform grid structure is centrally symmetrical, three situations can be used to represent all the tilting situations of the seed distributor. As shown in **Figure 7**, the three conditions are: horizontally tilted at 0° vertically tilted at 0° (*H*0°*V*0°), horizontally tilted at 5° vertically tilted at 0° (*H*5°*V*0°), and horizontally tilted at 5° vertically tilted at 5° (*H*5°*V*5°).

Response surface tests are essential for evaluating the impact of factors on performance (Zhang et al., 2024). *C*_1_, *C*_2_ and *C*_3_ were used to represent the variation coefficient of seed distributing uniformity in three situations of *H*0°*V*0°, *H*5°*V*0°, and *H*5°*V*5°, respectively. And were used as the evaluation index. A three-factor three-level Box-Behnken design (BBD) test was carried out with cone height *h*_1_, seed storage tube diameter *d* and seed storage tube lifting height *h*_2_ as the test factors. Based on the single factor test, the value ranges of each factor were determined as follows: the cone height *h*_1_ was 60-80 mm, the seed storage tube diameter *d* was 50-70 mm, and the seed storage tube lifting height *h*_2_ was 35-45 mm. The test factors and the levels are shown in **Table 3**. A total of 17 groups of tests were conducted, each group of tests was repeated 20 times, and the mean was taken as the test result.

**Table 3:** BBD test factors and levels.

Level	Cone height *h*_1_/mm	Seed storage tube diameter *d*/mm	Seed storage tube lifting height *h*_2_/mm
1	80	70	45
0	70	60	40
-1	60	50	35

### 2.7 Bench and field tests

**Figure 8:**
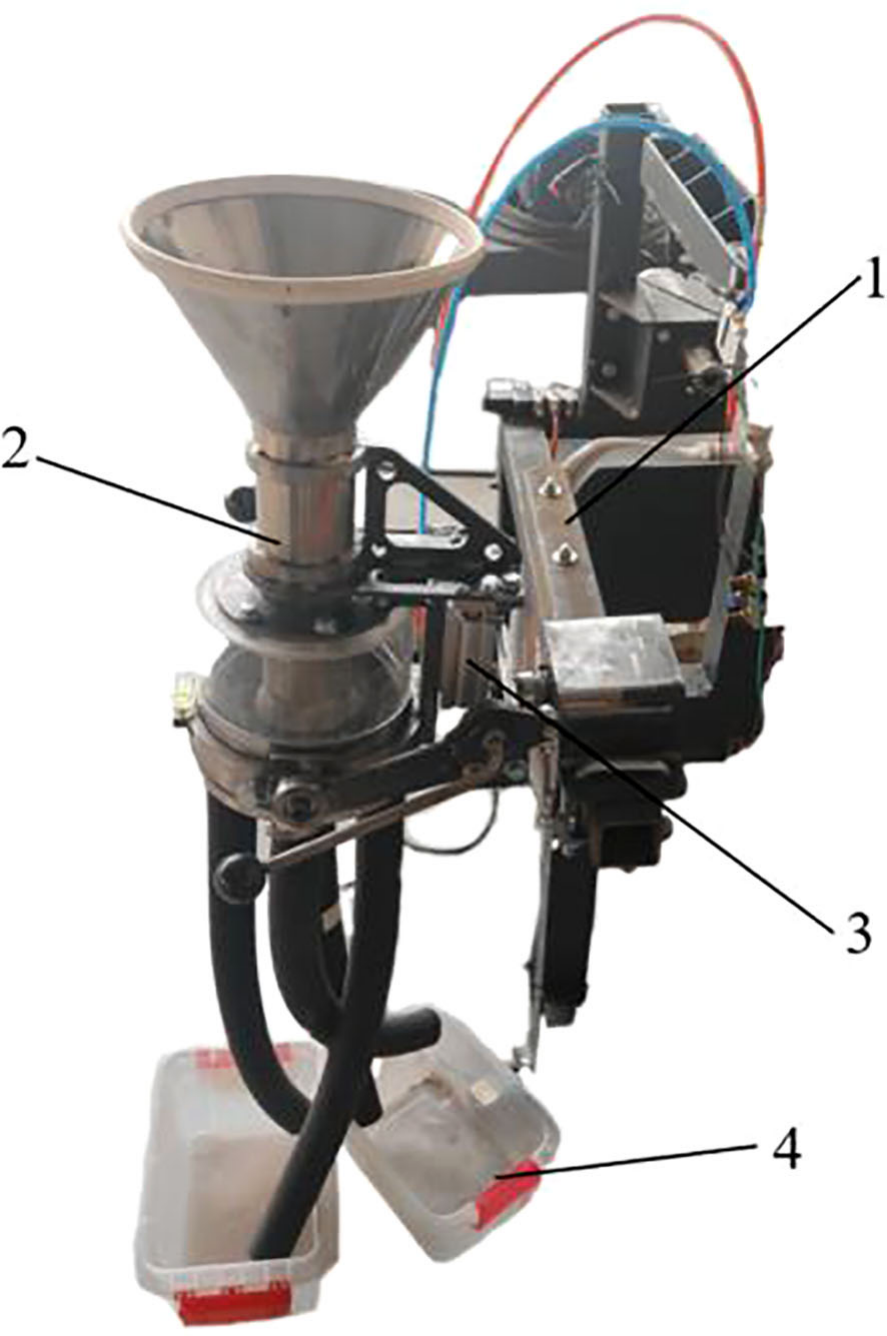
The pneumatic lifting seed distributor test bench. 1. Bench; 2. Pneumatic lifting seed distributor; 3. Pneumatic lifting mechanism; 4. Seed collection box.

In order to verify the accuracy of the optimization results and compare them with the traditional seed distributor, the pneumatic lifting seed distributor designed in this study was processed and a bench test was conducted. A total of 20 groups of the bench verification test were carried out, and the average value was taken as the test result. The test bench includes a bench, a pneumatic lifting seed distributor, a seed collection box and a corresponding control system, etc. The test equipment includes a grain counter and an inclinometer, etc. The test bench site is shown in **Figure 8**.

**Figure 9:**
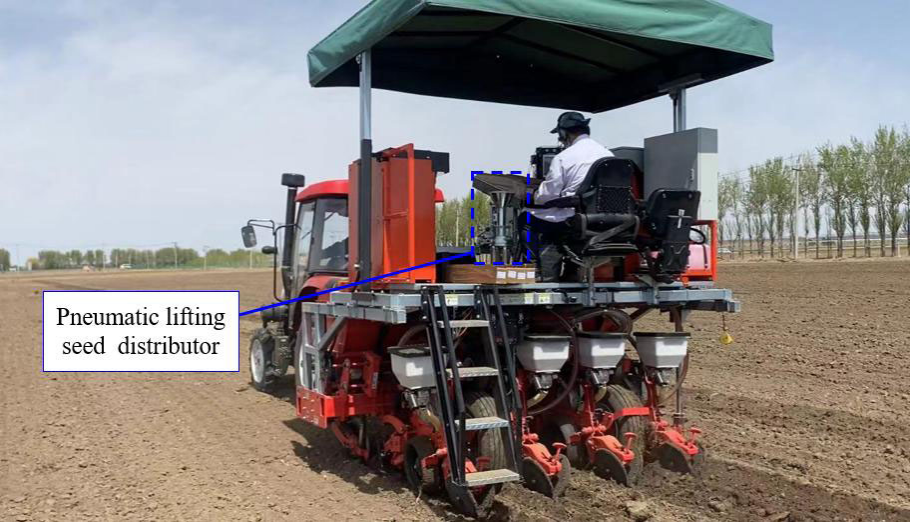
Field test.

In order to verify the operating performance of the pneumatic lifting seed distributor and compare it with the operating performance of the traditional cone uniform seed distributor, the prototype processing and assembly were completed based on the 2BY4XJ four-row maize plot seeder and the optimal parameters. In April 2024, a seed distribution performance test was conducted in the maize plot breeding test field of Syngenta Group Seed Company in Changchun City, Jilin Province. The test process is shown in **Figure 9**. The test plot area is 12 m ^2^ (row length 5 m, row spacing 0.6 m), and the plot spacing is 1 m. The plot seeder was equipped with two types of seed distributor and moved forward to 20 test plots, carrying out 20 seed distribution operations in total.

## 3 Results and discussion

### 3.1 Single factor simulation test results and analysis

#### 3.1.1 Effect of cone height *h*_1_ on seed distributing performance

**Figure 10:**
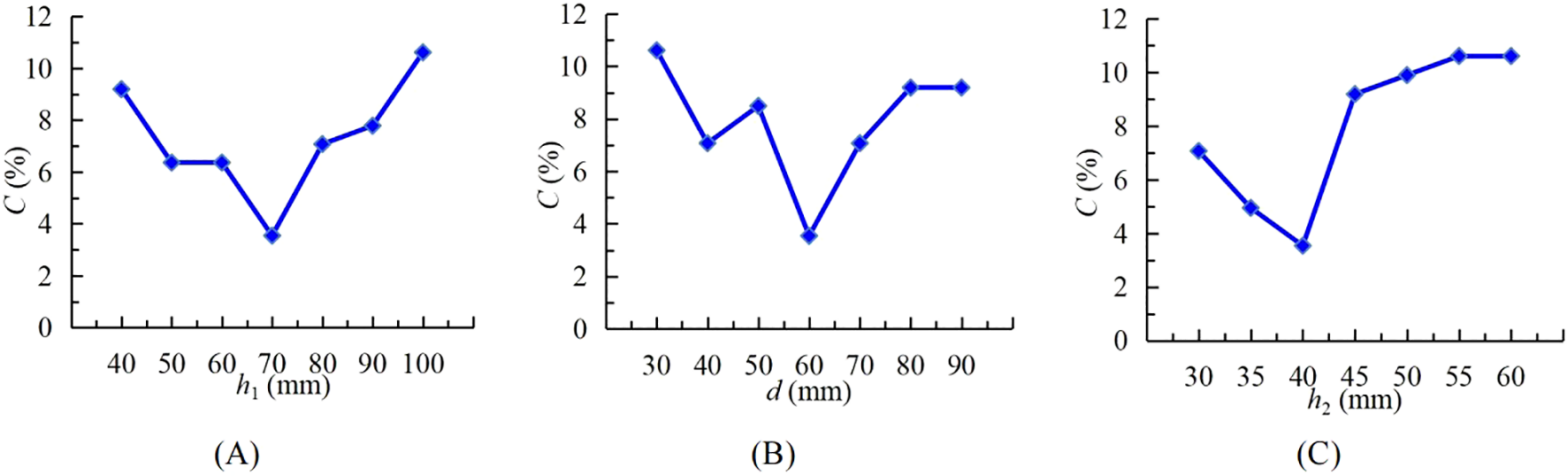
Effects of factors on variation coefficient of seed distributing uniformity *C.* **(A)** *H*0°*V*0°; **(B)** *H*5°*V*0°; **(C)** *H*5°*V*5°.

At *d* of 60 mm and *h*_2_ of 40 mm, single factor test was carried out with seven levels of *h*_1_ ranging from 40 mm to 100 mm. The test result is shown in **Figure 10A**. *C* (the variation coefficient of seed distributing uniformity) showed a trend of first decreasing and then increasing. When the *h*_1_ was low, the part of the cone located in the seed storage tube was small, and the seeds were randomly distributed in the seed storage tube. When the seed storage tube was lifted upward, the seeds on the cone fail to distribute in time and reach the uniform grid, resulting in an increase in *C*. When the *h*_1_ is high, the part of the cone located in the seed storage tube had made the seeds more evenly distributed. But with the increase of the *h*_1_, the seeds need more travel to reach the uniform grid. Meanwhile, the movement states of the seeds on the cone surface were relatively random. The seeds were easy to collide with the cone surface and bounce, deviating from the original movement trajectory, resulting in an increase in *C*. Therefore, *h*_1_ of 60~80 mm was selected as the multi-factor test range.

#### 3.1.2 Effect of seed storage tube diameter *d* on seed distributing performance

When *h*_1_ was 70 mm and *h*_2_ was 40 mm, seven levels within *d* ranged from 30 mm to 90 mm were selected for a single factor test. The test result was shown in **Figure 10B**. *C* showed an overall trend of first decreasing and then increasing. When *d* is small, most seeds were at a large distance from the cone in the vertical direction within the seed storage space. When the seed storage tube was lifted, the seeds that do not contact the cone were easy to collide with the cone surface and bounce. At this time, the seeds cannot slide down along the cone surface, and the cone lost its seed distribution function, resulting in an increase in *C*. When the *d* was large, the seeds in the seed storage tube were more distributed in the horizontal direction within the seed storage space, and most seeds did not contact the cone. When the seed storage tube was lifted, these seeds that do not contact the cone will also bounce, resulting in an increase in *C*. Therefore, *d* of 50~70 mm was selected as the multi-factor test range.

#### 3.1.3 Effect of seed storage tube lifting height *h*_2_ on seed distributing performance

In the preliminary test, it was found that when the seed storage tube was lifted too low (less than 30 mm). The seeds in the seed storage tube were easily stuck in the gap formed by the bottom of the seed storage tube and the cone, reducing the seed distributing uniformity (Yang, 2019b). The remaining seeds will be mixed with the next batch of seeds entering the seed storage tube, causing seed contamination and the failure of the breeding test. Under the conditions of *h*_1_ of 70 mm and *d* of 60 mm, a single factor test was conducted with seven levels of *h*_2_ ranging from 30 mm to 60 mm. The test results are shown in **Figure 10C**. *C* showed a trend of first decreasing, then increasing and finally leveling off. When *h*_2_ was low, *C* was larger. When *h*_2_ exceeds 45 mm, *C* did not change significantly due to the increase in *h*_2_. This is because when *h*_1_ and *d* are fixed, the seed population height in the seed storage space is determined. When *h*_2_ exceeded the seed population height, it would not have a significant impact on the seed distributing effect. Therefore, *h*_2_ of 35~45 mm was selected as the multi-factor test range.

### 3.2 Results and analysis of BBD test

#### 3.2.1 Significance test and regression model

The test design and results are shown in **Table 4**. The analysis of variance (ANOVA) of the variation coefficient of seed distributing uniformity was shown in **Table 5**. For the regression model of *C*_1_, the influences of *X*_2_, *X*_3_, *X*_1_ *X*_2_, *X*_1_ *X*_3_, *X*_2_ *X*_3_, *X*_1_^2^ and *X*_3_^2^ on the model were extremely significant, while the other items were not significant. The simplified regression model was extremely significant and the lack of fit term was not significant, so the regression model was valid. After eliminating the non-significant terms except *X*_1_, the regression equation was obtained, as shown in Equation 6.

**Table 4:** Test design and results.

Test No.	*X*1	*X*2	*X*3	*C*_1_ (%)	*C*_2_ (%)	*C*_3_ (%)
1	-1	-1	0	6.36	9.90	4.95
2	1	-1	0	7.78	5.66	6.36
3	-1	1	0	5.83	5.48	9.90
4	1	1	0	4.95	8.72	5.13
5	-1	0	-1	7.78	5.66	8.49
6	1	0	-1	6.72	8.31	7.07
7	-1	0	1	5.13	9.90	8.66
8	1	0	1	6.89	6.54	7.25
9	0	-1	-1	8.31	6.36	7.43
10	0	1	-1	4.95	8.49	8.66
11	0	-1	1	5.83	10.61	6.72
12	0	1	1	5.30	6.19	8.66
13	0	0	0	5.13	7.25	5.66
14	0	0	0	5.13	7.07	6.36
15	0	0	0	5.66	8.05	6.72
16	0	0	0	5.48	6.36	6.89
17	0	0	0	6.01	7.78	7.07

**Table 5:** Analysis of variance for variation coefficient of seeding uniformity.

Source	*C*_1_		*C*_2_		*C*_3_	
	*F* value	*P* value	*F* value	*P* value	*F* value	*P* value
Model	22.65	0.0002**	16.14	0.0007**	14.78	0.0009**
*X*1	2.20	0.1817	1.36	0.2821	23.03	0.0020**
*X*2	75.16	< 0.0001**	6.19	0.0418*	28.53	0.0011**
*X*3	30.39	0.0009**	9.07	0.0196*	0.078	0.7882
*X*1X2	15.13	0.0060**	51.96	0.0002**	45.91	0.0003**
*X*1X3	22.74	0.0020**	33.55	0.0007**	1.20E-04	0.9916
*X*2X3	22.90	0.0020**	39.84	0.0004**	0.61	0.4618
*X*12	19.74	0.0030**	0.12	0.7437	0.01	0.9222
*X*22	0.56	0.4789	0.78	0.4051	0.01	0.9222
*X*32	12.42	0.0097**	2.34	0.1702	34.47	0.0006**
Lack of fit	0.13	0.9386	0.12	0.9453	0.23	0.8721

**highly significant (*P ≤* 0.01); *significant (0.01<*P ≤* 0.05).


(6) $$C_{1}=5.48+0.16X_{1}-0.91X_{2}-0.58X_{3}-0.58X_{1}X_{2}+0.71X_{1}X_{3}+0.71X_{2}X_{3}+0.64X_{1}^{2}+0.51X_{3}^{2}$$

For the regression model of *C*_2_, the influences of *X*_1_ *X*_2_, *X*_1_ *X*_3_ and *X*_2_ *X*_3_ on the model were extremely significant, *X*_2_ and *X*_3_ on the model were significant, while the other items were not significant. The simplified regression model was extremely significant and the lack of fit term was not significant, so the regression model was valid. After eliminating the non-significant terms except *X*_1_, the regression equation was obtained, as shown in Equation 7.


(7) $$C_{2}=7.30-0.21X_{1}-0.46X_{2}+0.55X_{3}+1.87X_{1}X_{2}-1.50X_{1}X_{3}-1.64X_{2}X_{3}$$

For the regression model of *C*_3_, the influences of *X*_1_, *X*_2_, *X*_1_ *X*_2_, and *X*_3_^2^ on the model were extremely significant, while the other items were not significant. The simplified regression model was extremely significant and the lack of fit term was not significant, so the regression model was valid. After eliminating the non-significant terms except *X*_3_, the regression equation was obtained, as shown in Equation 8.


(8) $$C_{3}=6.54-0.77X_{1}+0.86X_{2}-0.045X_{3}-1.55X_{1}X_{2}+1.31X_{3}^{2}$$

#### 3.2.2 Response surface analysis of each factor to the test index

**Figure 11:**
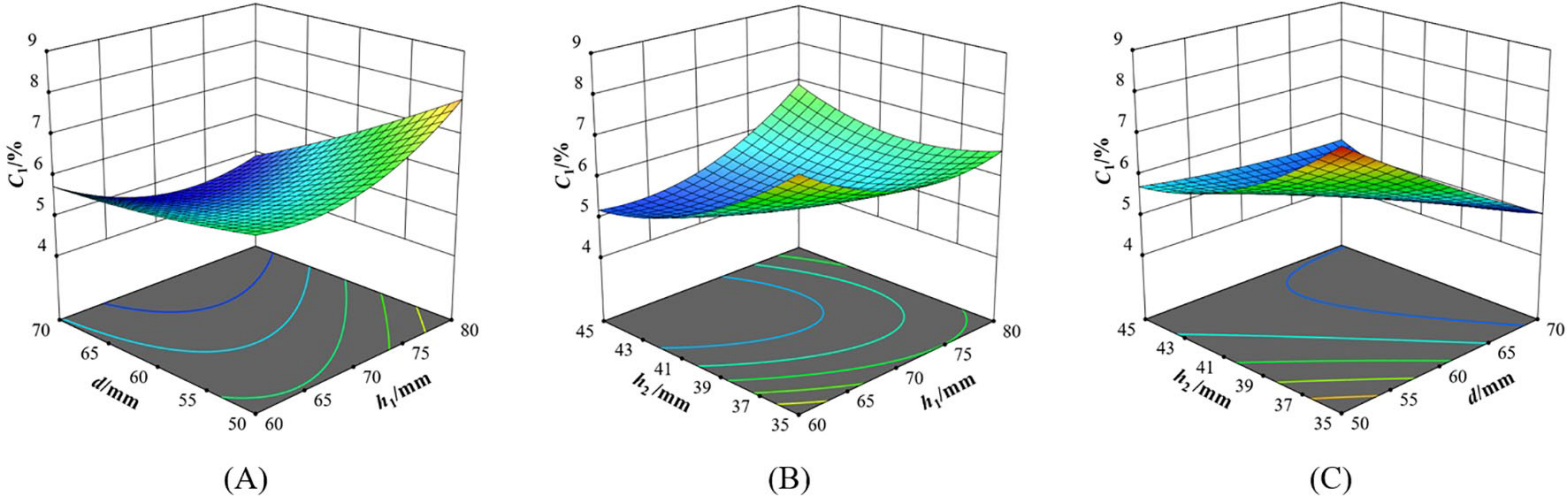
Interaction of factors on variation coefficient of seed distributing uniformity *C*_1_. **(A)** *C*_1_=*f*(*h*_1_, *d*, 40); **(B)** *C*_1_=*f*(*h*_1_, 60, *h*_2_); **(C)** *C*_1_=*f*(70, *d*, *h*_2_).

**Figure 12:**
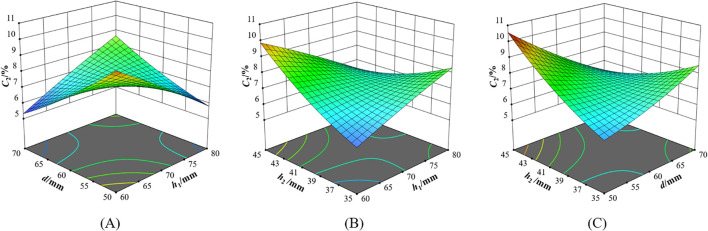
Interaction of factors on variation coefficient of seed distributing uniformity **(A)** *C_2_=f(h_1_, d, 40)*; **(B)** *C_1_=f(h_1_, 60, h_2_)*; **(C)** *C_1_=f(70, d, h_2_)*.

**Figure 13:**
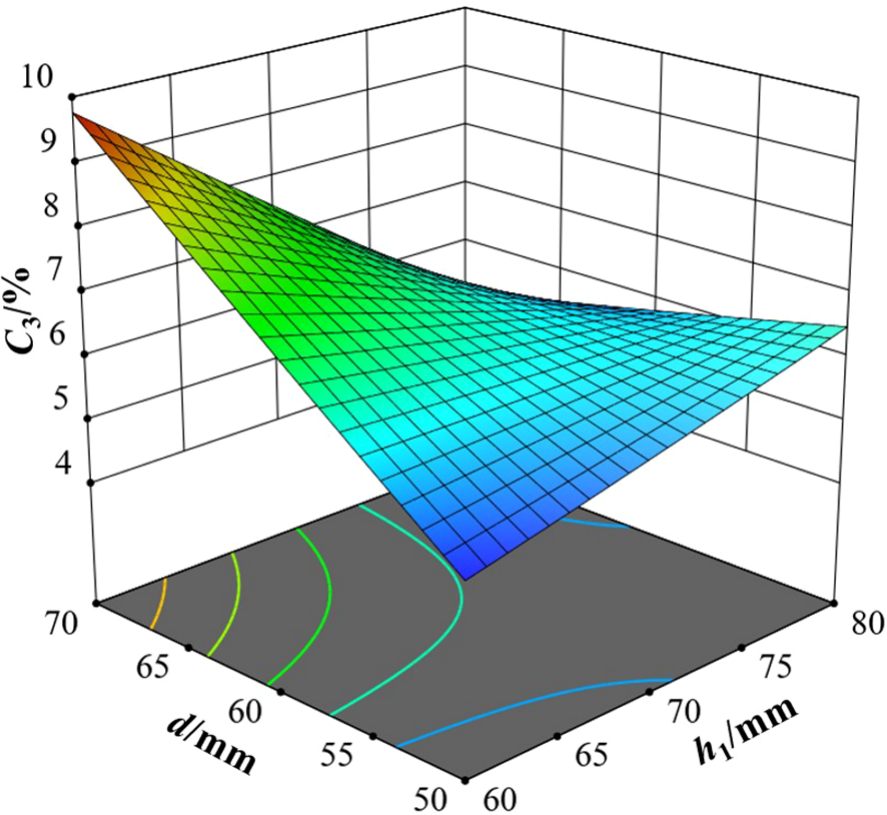
Interaction of factors on variation coefficient of seed distributing uniformity *C*_3_.

The interactive effects of various factors on the variation coefficient of seed distributing uniformity are shown in **Figures 11**–**13**.

When the seed distributor was in *H*0°*V*0°, it can be seen from **Figure 11A** that when *h*_2_ was 40 mm, if *h*_1_ was fixed, *C*_1_ decreased with the increase of *d*. If *d* was less than 60 mm, *C*_1_ increased with the increase of *h*_1_. If *d* was greater than 60 mm, *C*_1_ decreased slightly with the increase of *h*_1_. This shows that the cone played the role in early seed distribution, making the distribution of seeds in the seed storage space more uniform (Yang et al., 2013). In the case of a larger *d*, the chances of seeds slipping along the cone were greater. It can be seen from **Figure 11B** that when *d* was 60 mm, if *h*_1_ was less than 70 mm, *C*_1_ decreased with the increase of *h*_2_. If *h*_1_ was greater than 70 mm, *C*_1_ increased with the increase of *h*_2_. If *h*_2_ was less than 40 mm, *C*_1_ decreased with the increase of *h*_1_. If *h*_2_ was greater than 40 mm, *C*_1_ increased with the increase of *h*_1_. This is because the lower *h*_2_ can limit the movement of seeds in the seed storage space in the horizontal direction. This increased the chances of the seeds coming into contact with the cone. On the whole, with the increase of *h*_1_ and *h*_2_, *C*_1_ showed a trend of first decreasing and then increasing. It can be seen from **Figure 11C** that when *h*_1_ was 70 mm, if *d* was less than 60 mm, *C*_1_ decreased with the increase of *h*_2_. If *d* was greater than 60 mm, *C*_1_ did not change significantly with the increase of *h*_2_. If *h*_2_ was less than 40 mm, *C*_1_ increased with the increase of *d*. If *h*_2_ was greater than 40 mm, *C*_1_ does not change significantly with the increase of *d*. This indicates that when *d* was large, lowering *h*_2_ cannot effectively restrict the horizontal movement of seeds. This resulted in more collisions between seeds and the cone, reducing the seed distributing uniformity.

When the seed distributor was in *H*5°*V*0°, it can be seen from **Figure 12A** that when *h*_2_ was 40 mm, if *h*_1_ was less than 70 mm, *C*_2_ decreased with the increase of *d*. If *h*_1_ was greater than 70 mm, *C*_2_ increased with the increase of *d*. If *d* was less than 60 mm, *C*_2_ decreased with the increase of *h*_1_; if *d* was greater than 60 mm, *C*_2_ increased with the increase of *h*_1_. With the increase of *h*_1_ and *d*, *C*_2_ showed an overall trend of first decreasing and then increasing. The larger *h*_1_ allowed the seeds to be distributed earlier and more evenly. However, when the seed distributor was tilted, the seeds in the seed storage space moved to the lower end of the grid. This resulted in uneven distribution of seeds entering the two Y-shaped seed guiding tubes. It can be seen from **Figure 12B** that when *d* was 60 mm, if *h*_1_ was less than 70 mm, *C*_2_ increased with the increase of *h*_2_. If *h*_1_ was greater than 70 mm, *C*_2_ decreased with the increase of *h*_2_. If *h*_2_ was less than 40 mm, *C*_2_ increased with the increase of *h*_1_. If *h*_2_ was greater than 40 mm, *C*_2_ decreased with the increase of *h*_1_. The smaller *h*_1_ allowed the seeds in the seed storage space to be concentrated on one side when the seed distributor was tilted. After the seed storage tube was lifted, the seeds enter the grid before they can be evenly distributed, resulting in a sharp increase in the variation coefficient of seed distribution uniformity. It can be seen from **Figure 12C** that when *h*_1_ was 70 mm, if *d* was less than 60 mm, *C*_2_ increased sharply with the increase of *h*_2_. If *d* was greater than 60 mm, *C*_2_ decreased with the increase of *h*_2_. If *h*_2_ was less than 40 mm, *C*_2_ increased with the increase of *d*. If *h*_2_ was greater than 40 mm, *C*_2_ decreased sharply with the increase of *d*.

When the seed distributor was in *H*5°*V*5°, it can be seen from **Figure 13** that when *h*_2_ was 40 mm, if *h*_1_ was less than 70 mm, *C*_3_ increased sharply with the increase of *d*. If *h*_1_ was greater than 70 mm, it decreased slightly with the increase of *d*. If *d* was less than 60 mm, *C*_3_ increased slightly with the increase of *h*_1_. If *d* was greater than 60 mm, *C*_3_ decreased sharply with the increase of *h*_1_.

When *h*_1_ was higher, the seed distributing effect was less affected by the tilt angle. No matter which direction the seed distributor was tilted, the cone can distribute the seeds evenly in advance. The seeds fell into the bottom of the seed storage space and were evenly distributed to four uniform grids. When the cone was too low, it is easy to cause the seeds to pile up on one side in the seed storage space. Most of the piled seeds fell into the same uniform grid without being evenly distributed, resulting in a decrease in the seed distributing effect. The larger *d*, the more seeds will accumulate in the inclined direction due to gravity. At this time, if *h*_1_ was low, the cone lost its seed distribution function, and the seeds will spread irregularly at the bottom of the seed storage space. The number of seeds falling into the uniform grid will change randomly, and it is impossible to find its inherent law.

#### 3.2.3 Parameter optimization

In order to obtain the optimal parameter combination of the seed distributor, the regression model of the test index was optimized and solved, with the lowest variation coefficient of seed distributing uniformity as the optimization target. According to the value range of each factor, the objective function and constraint conditions are shown in Equation 9. In the early tests, it was found that the tilt angle of the seed distributor was always fluctuating, so the weights of the three indicators *C*_1_, *C*_2_, and *C*_3_ were evenly distributed to ensure better seed distributing effect.

After solving, it was found that the seed distributor had the best seed separation effect when the *h*_1_ was 77 mm, *d* was 70 mm and *h*_2_ was 42 mm. At this time, the predicted variation coefficients of seed distributing uniformity *C*_1_, *C*_2_, and *C*_3_ were 5.03%, 7.39%, and 6.07%, respectively.


(9) $$\left\{ \begin{array}{l} \min C_{1}\left(h_{1},d,h_{2}\right)\\ \min C_{2}\left(h_{1},d,h_{2}\right)\\ \min C_{3}\left(h_{1},d,h_{2}\right)\\ \text{s}.\text{t}.\{\begin{array}{c} 60\text{mm}\leq h_{1}\leq 80\text{mm}\\ 50\text{mm}\leq d\leq 70\text{mm}\\ 35\text{mm}\leq h_{2}\leq 45\text{mm} \end{array} \end{array}\right.$$

### 3.3 Operational performance of bench and field tests

**Figure 14:**
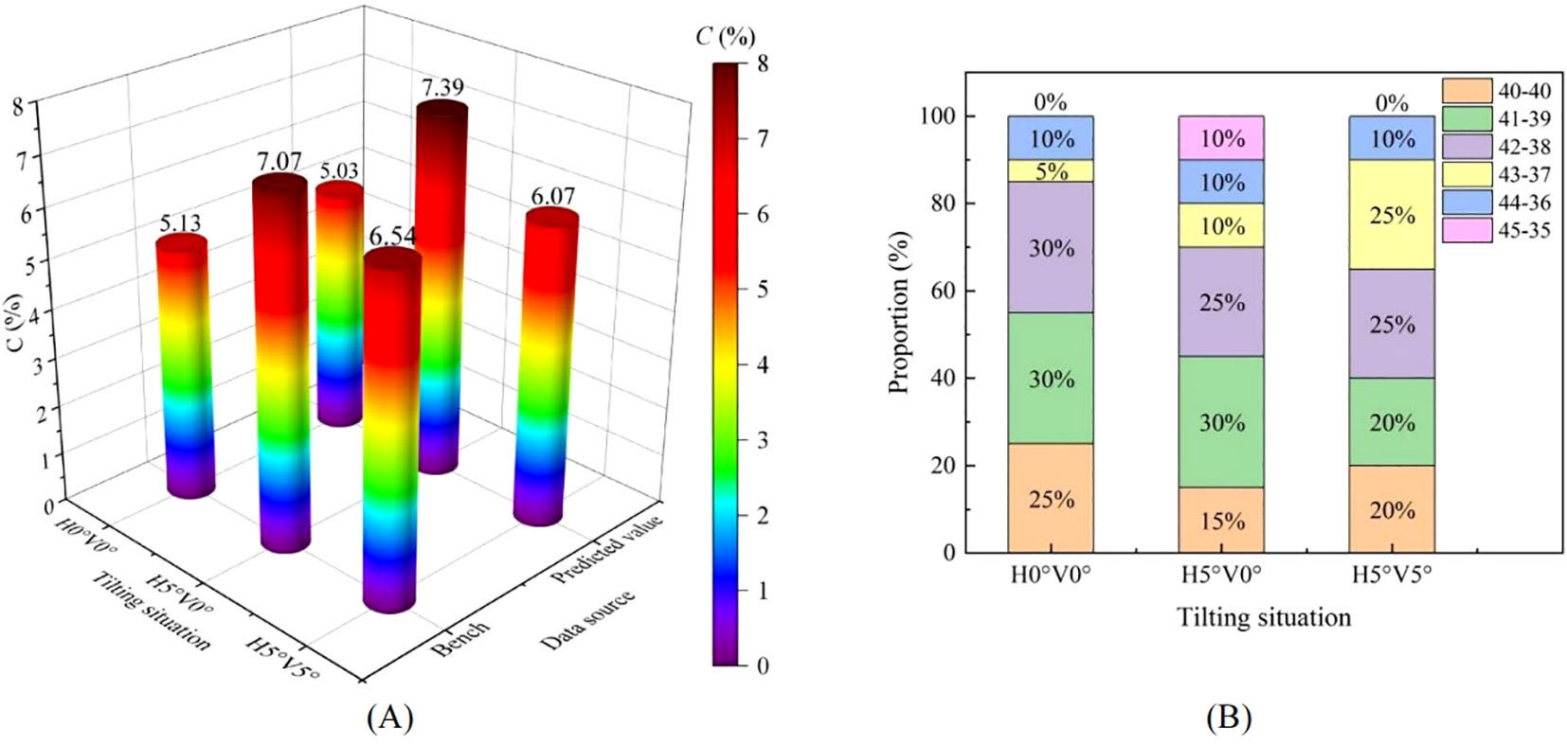
The results of bench. **(A)** Comparison table between simulation and predicted values; **(B)** The proportion of different situations.

The bench test results are shown in **Figure 14**. The bench test results were basically consistent with the predicted values. The differences in the three tilt situations were 0.10, 0.32 and 0.47, respectively. The variation coefficients of seed distributing uniformity of the pneumatic lifting seed distributor were between 5.13% and 7.07%. When the seed distributor was in *H*0°*V*0°, the seeds were evenly distributed into two Y-shaped seed guide tubes, and the seed distributing effect was best. The situation where both Y-shaped seed guide tubes had 40 seeds flowing out accounted for 25%. When the seed distributor was in *H*5°*V*0°, most of the seeds were located above one uniform grid. The difference in the number of seeds flowing into the two Y-shaped seed guide tubes increased. At this time, the seed distributing effect was the worst. When the seed sorting device was in *H*5°*V*5°, most of the seeds were located in the middle of two uniform grids, and finally flow into their respective Y-shaped seed guide tubes. The seed distributing effect in this situation was better than that when it was in *H*5°*V*0°, but worse than when the seed sorting device was in *H*0°*V*0°. The order of variation coefficients of seed distributing uniformity in the three tilt situations was: *H*0°*V*0°, *H*5°*V*5° and *H*5°*V*0°. When the tilt angle of the seed distributor was in the range of 0~5°, the maximum difference of the variation coefficient of seed distributing uniformity was 1.94%. Meanwhile, it can be considered that there was basically no effect on the seed distributing effect.

**Figure 15:**
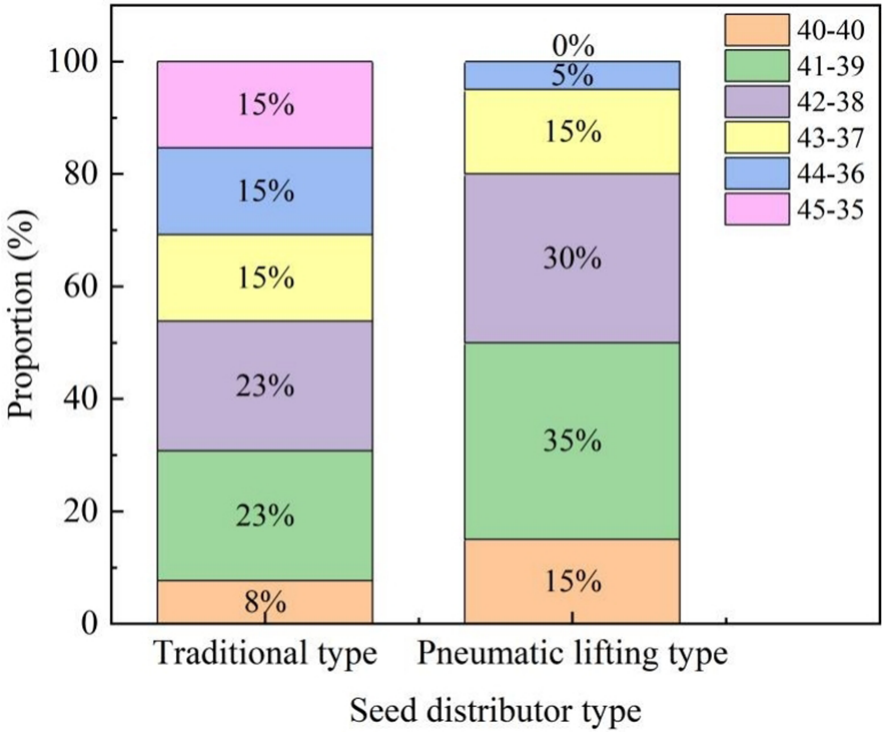
The proportion of different situations in field.

The field test results are shown in **Figure 15**. The number of seeds flowing out of the two Y-shaped seed guide tubes of the pneumatic lifting seed distributor were 40-40 and 41-39, which were more than that of the traditional seed distributor. The comparison of variation coefficient of seed distributing uniformity is shown in **Table 6**. The variation coefficient of seed distributing uniformity in the field test was 5.66%, which was within the range of 5.13% to 7.07% of the variation coefficient of seed distributing uniformity in the bench test results. The accuracy of the bench test had been verified. Compared with the traditional seed distributor, the variation coefficient of seed distributing uniformity of the pneumatic lifting seed distributor reduced by 1.23 percentage points. The test results showed that the pneumatic lifting seed distributor can achieve better seed distributing operation without leveling, and there was no need to configure an additional leveling mechanism.

**Table 6:** Comparison of the operational effects of seed distributors.

Seed distributor	The variation coefficients of seed distributing uniformity *C*/%
Pneumatic lifting type	5.66 ± 0.22
Traditional type	6.89 ± 0.14
Difference	1.23

### 3.4 Uniform seed distribution for sustainable phytoprotection

Plot breeding tests basically run through the entire process of developing various excellent seed varieties (Shang et al., 2021). Seed production is an important link in the modern agricultural production process and plays a vital role in the sustainable development of agriculture. Its quality directly affects the yield and quality of crops. Maintaining soil fertility and preventing agricultural residues both require good quality seeds and efficient production techniques. Scientific seed management also plays an important role in preventing and controlling pests and diseases, reducing pesticide use, and reducing environmental pollution.

Efficient and high-quality seed distribution can improve the seeding performance of the plot seeder. The pneumatic lifting seed distributor designed in this paper can effectively ensure the uniformity of seed distribution when the seeder is in an inclined state. It can improve the working efficiency and quality of the seeder. The quality of plot seeder operation has a significant impact on the consistency and health of the crop during its early growth stages. It can ensure that the seedlings are neat and uniform, which is convenient for later field management. At the same time, high-quality seeds can be promoted for production, production standards can be ensured, and production processes can be reduced. Uniform seed distribution combined with precise seeding and the use of intelligent technology form a structure with neat rows and columns. This can provide help in optimizing pesticide application strategies. When necessary, infected areas can be targeted more accurately for local treatment rather than spraying large areas with chemicals. This reduces the amount of chemicals used and their impact on non-target organisms.

## 4 Conclusion

This study designed a new type of pneumatic lifting seed distributor, which directly drove the seed storage tube to lift up through the air cylinder. The situation where the seed storage tube got stuck when moving up and down was avoided. The optimized seed distributor had basically no effect on the seed distributing operation effect when it was tilted.

1. The movement state and force of the maize seed sliding, rolling and bouncing on the cone were analyzed. It was concluded that the maize seed sliding along the cone was the best motion state to play the role of cone seed distribution, which could improve the seed distribution uniformity of the seed distributor.
2. The structure and operational parameters of the pneumatic lifting seed distributor were optimized through single factor tests and BBD test. The optimal parameter combination for seed distributing effect was obtained: *h*_1_ was 77 mm, *d* was 70 mm, and *h*_2_ was 42 mm. The bench verification tests were carried out with the optimal parameter combination. When the seed distributor were in *H*0°*V*0°, *H*5°*V*0°, and *H*5°*V*5°, the variation coefficients of seed distributing uniformity were 5.13%, 7.07%, and 6.54%, respectively, which were basically consistent with the predicted values. When the titling angle of the seed distributor was in the range of 0~5°, the maximum difference of the variation coefficients of seed distributing uniformity was 1.94%.
3. Through field tests, the pneumatic lifting seed distributor was compared with the traditional seed distributor. The results showed that the variation coefficient of the pneumatic lifting seed distributor was 5.66%, which was 1.23 percentage points lower than that of the traditional seed distributor. The pneumatic lifting seed distributor designed in this study had better seed distributing effect than the traditional seed distributor.

## Data Availability

The original contributions presented in the study are included in the article/supplementary material. Further inquiries can be directed to the corresponding author.

## References

[B1] Cheng X. Li H. Wang Q. He J. Lu C. Yang W. (2019). Design and experiment of wheat seeding control system in plot seede. Trans. Chin. Soc. Agric. Machinery 50, 30–38. doi: 10.6041/j.issn.1000-1298.2019.07.003

[B2] Dong J. Zhang S. Zheng Z. Wu J. Huang Y. Gao X. (2024). Development of a novel perforated type precision metering device for efficient and cleaner production of maize. J. Cleaner Product 443, 140928. doi: 10.1016/j.jclepro.2024.140928

[B3] Dun G. Mao N. Liu W. Wu X. Zhou C. Ji W. (2022). Design and experiment of four-bar translational seed metering device for soybean plot breeding. Trans. Chin. Soc. Agric. Machinery 53, 70–78. doi: 10.6041/j.issn.1000-1298.2022.04.007

[B4] Gao X. Cui T. Zhou Z. Yu Y. Xu Y. Zhang D.. (2021). DEM study of particle motion in novel high-speed seed metering device. Advanced Powder Technol. 32, 1438–1449.30. doi: 10.1016/j.apt.2021.03.002

[B5] Gao X. Xie G. Li J. Shi G. Lai Q. Huang Y. (2023). Design and validation of a centrifugal variable-diameter pneumatic high-speed precision seed-metering device for maize. Biosyst. Eng. 227, 161–181. doi: 10.1016/j.biosystemseng.2023.02.004

[B6] Gao X. Xie G. Xu Y. Yu Y. Lai Q. (2022). Application of a staggered symmetrical spiral groove wheel on a quantitative feeding device and investigation of particle motion characteristics based on DEM. Powder Technol. 407, 117650. doi: 10.1016/j.powtec.2022.117650

[B7] Gong L. Yuan Y. Shang S. Jiang J. Zheng Y. (2011). Design and experiment on electronic control system for plot seeder. Trans. Chin. Soc. Agric. Eng. 27, 122–126. doi: 10.3969/j.issn.1002-6819.2011.05.020

[B8] Hu H. Zhou Z. Wu W. Yang W. Li T. Chang C.. (2021). Distribution characteristics and parameter optimisation of an air-assisted centralised seed-metering device for rapeseed using a CFD-DEM coupled simulation. Biosyst. Eng. 208, 246–259. doi: 10.1016/j.biosystemseng.2021.06.005

[B9] Lei X. Liao Y. Zhang Q. Wang L. Liao Q. (2018). Numerical simulation of seed motion characteristics of distribution head for rapeseed and wheat. Comput. Electron. Agric. 150, 98–109. doi: 10.1016/j.compag.2018.04.009

[B10] Liu S. Shang S. Yang R. Zheng Y. Wang Y. Li Q. (2011a). Design of automatic supplying seed system for plot seeder of rape. Trans. Chin. Soc. Agric. Machinery 42, 91–95. doi: 10.3969/j.issn.1000-1298

[B11] Liu S. Shang S. Yang R. Zheng Y. Wang Y. Zhao D.. (2011b). Test on performance of seed-filling device of plot seeder. J. China Agric. Univ. 16, 156–163. doi: 10.11841/j.issn.1007-4333.2011.03.027

[B12] Liu S. Shang S. Yang R. Zheng Y. Zhao D. Zhao J. (2010). Test and optimization of parameters for the storing device of plot seeder. Trans. Chin. Soc. Agric. Eng. 26, 101–108. doi: 10.3969/j.issn.1002-6819.2010.09.016

[B13] Markauskas D. Ramírez-Gómez Á. Kačianauskas R. Zdancevičius E. (2015). Maize grain shape approaches for DEM modelling. Comput. Electron. Agric., 118247–118258. doi: 10.1016/j.compag.2015.09.004

[B14] Mudarisov S. Badretdinov I. Rakhimov Z. Lukmanov R. Nurullin E. (2020). Numerical simulation of two-phase “Air-Seed” flow in the distribution system of the grain seeder. Comput. Electron. Agric. 168, 105151. doi: 10.1016/j.compag.2019.105151

[B15] Shang S. Wu X. Yang R. Li G. Yang X. Chen D. (2021). Research status and prospect of plot-sowing equipment and technology. Trans. Chin. Soc. Agric. Machinery 52, 1–20. doi: 10.6041/j.issn.1000-1298.2021.02.001

[B16] Shang S. Yang R. Yin Y. Guo P. Sun Q. (2010). Current situation and development trend of mechanization of field experiments. Trans. Chin. Soc. Agric. Eng. 26, 5–8. doi: 10.3969/j.issn.1002-6819.2010.z1.002

[B17] Song Q. (2019). Development of Precision Planter in maize Community. Jilin Agricultural University, Changchun.

[B18] Yang W. (2019b). Research on Technology and Equipment of Precision Seeding in Maize Breeding. Chinese Academy of Agricultural Mechanization Sciences, Beijing.

[B19] Yang W. Fang X. Li J. Li L. (2019a). Design and experiment of air-suction precision seed meter with self-clearing seed chamber for maize plot test. Trans. Chin. Soc. Agric. Machinery 50, 64–73. doi: 10.6041/j.issn.1000-1298.2019.06.007

[B20] Yang C. Shang S. Yang R. Wang C. (2015). Theoretical study on the cone-centrifugal seed metering device. J. Agric. Mechanization Res. 37, 63–66. doi: 10.13427/j.cnki.njyi.2015.06.015

[B21] Yang S. Yin M. Zhang S. (2013). Experimental research of the grass seed motion and distribuion uniformity on distributor cone. J. China Agric. Univ. 18, 178–184. doi: 10.11841/j.issn.1007-4333.2013.03.026

[B22] Yang R. Zhang X. Li J. Shang S. Chai H. (2016). Parameter optimization and experiment on cone canvas belt type seed-metering device. Trans. Chin. Soc. Agric. Eng. 32, 6–13. doi: 10.11975/j.issn.1002-6819.2016.03.002

[B23] Yu C. Chen Z. Chen L. (2023). Design and test of electronic control seeding system for wheat plot drill. Trans. Chin. Soc. Agric. Machinery 54, 75–83. doi: 10.6041/j.issn.1000-1298.2023.01.008

[B24] Zhang T. Shang S. Wang D. (2017). Design of six lines synchronous plot seeder. J. Agric. Mechanization Res. 39, 144–149. doi: 10.13427/j.cnki.njyi.2017.05.027

[B25] Zhang M. Zhao P. Gao X. Lai Q. (2024). Development of a new environmentally friendly and efficient centrifugal variable diameter metering device. Front. Plant Sci. 15. doi: 10.3389/fpls.2024.140420 PMC11251964 39022608

[B26] Zhao P. Gao X. Su Y. Xu Y. Huang Y. (2024). Investigation of seeding performance of a novel high-speed precision seed metering device based on numerical simulation and high-speed camera. Comput. Electron. Agric. 217, 108563. doi: 10.1016/j.compag.2023.108563

[B27] Zhou L. (2021). DEM-based Modelling of Maize Seeds and the Simulation Analysis and Experimental Study of the Seed-Sowing. Jilin University, Changchun.

[B28] Zhu M. Chen H. Li Y. (2015). Investigation and development analysis of seed industry mechanization in China. Trans. Chin. Soc. Agric. Eng. 31, 1–7. doi: 10.11975/j.issn.1002-6819.2015.14.001

